# Endoscopic-Ultrasound-Guided Tissue Sampling Facilitates the Detection of Local Recurrence and Extra Pelvic Metastasis in Pelvic Urologic Malignancy

**DOI:** 10.1155/2012/219521

**Published:** 2012-06-19

**Authors:** Ferga C. Gleeson, Jonathan E. Clain, R. Jeffrey Karnes, Elizabeth Rajan, Mark D. Topazian, Kenneth K. Wang, Michael J. Levy

**Affiliations:** ^1^Division of Gastroenterology & Hepatology, Mayo Clinic, Rochester, MN 55905, USA; ^2^Department of Urology, Mayo Clinic, Rochester, MN 55905, USA

## Abstract

Pelvic lymph node dissection is the gold standard for assessing nodal disease in prostate or bladder cancer and is superior to CT, MRI and PET staging. Endoscopic ultrasound (EUS) provides an alternative, less invasive method of cytohistologic material acquisition, but its performance in pelvic urologic malignancy is unknown. Therefore, our aim was to evaluate the diagnostic accuracy of EUS guided tissue sampling for these malignancies when compared to a composite cytohistologic and surgical gold standard. A median of 3 FNA passes were performed (*n* = 19 patients) revealing a sensitivity, specificity, PPV and NPV of 94.4% (72–99), 100% (2–100), 100% (80–100) and 50% (1–98) respectively. The perirectal space was the most frequently sampled location irrespective of the primary urological cancer origin. Final diagnosis established by EUS tissue sampling included bladder cancer (*n* = 1), bladder cancer local recurrence (*n* = 8), bladder cancer extra pelvic metastases (*n* = 1), prostate cancer (*n* = 2), prostate cancer local recurrence (*n* = 4), prostate cancer extra pelvic metastases (*n* = 1), testicular cancer extra pelvic metastases (*n* = 1) and a benign seminal vesicle (*n* = 1). EUS guided sampling of the gut wall, lymph nodes, or perirectal space yields suitable diagnostic material to establish the presence of primary, local recurrence or extra pelvic metastases of pelvic urologic malignancy.

## 1. Introduction

Regional lymph node assessment is important to estimate prognosis and to determine appropriate treatment in patients with urological cancer. The gold standard for assessing nodal status in prostate or bladder cancer is pelvic lymph node dissection, which is superior to CT, MRI, or PET given the poor predictive value of nodal-size criteria [1–3]. A recent meta-analysis evaluated the diagnostic accuracy of CT and MRI for staging pelvic lymph nodes in patients with prostate cancer and found the specificity to be only 82% for both CT and MRI, while the sensitivities were 42% and 39%, respectively [4]. Similarly, a study evaluating EUS for locoregional staging of prostate cancer revealed a N1 sensitivity and specificity of 58.3% and 52.5%, respectively, using solely a size criterion of 10 mm [5]. However, isolated EUS FNA cases of mediastinal and iliac vessel lymph node metastasis have been reported in prostate cancer evaluation [6, 7]. In addition, EUS FNA has been used to document prostate or bladder cancer recurrence manifesting as rectal linitis plastica [8–10]. EUS may also provide a therapeutic role by guiding drainage of pelvic fluid collections and with fiducial placement to guide radiation therapy for prostate cancer recurrence [11–16].

The aim of this retrospective case series was to evaluate the potential utility of EUS FNA and TCB to facilitate the detection of potential sites of local and distant metastasis.

## 2. Patients and Methods

A prospectively maintained EUS database was reviewed to identify patients who had undergone FNA or TCB to evaluate pelvic urological disease cancer of the bladder, prostate, and testicle, excluding renal cancer, from January 1, 2002 to June 30, 2011. Patient clinical, radiologic, EUS FNA-TCB, cytopathology, histopathology, and immune-staining data were abstracted by chart review. Cytological and histological samples were acquired under EUS FNA or TCB guidance with a linear echoendoscope (Olympus GF-UC140P-AL5 or Olympus GF-UC 160P-AT8) using either a 22G (Echotip-22ECHO 3-22, Cook Endoscopy, Winston-Salem, NC, USA) or TCB needle (Tru-Cut needle Cook Quick-Core, Cook Endoscopy, Winston-Salem, NC, USA). Informed consent was obtained for all procedures described in this paper and the IRB granted study approval for this study.

## 3. Results

During the study period, 678 lower GI EUS FNA procedures were performed, of which 16 (2.6%) were to evaluate a suspected urological malignancy. An additional 3 upper GI EUS FNA cases were identified. Collectively, 19 patients (18 : 1, M : F) (age 67 ± 15.6 years) were identified who had undergone EUS FNA ± TCB to evaluate possible prostate, bladder, or testicular cancer. All patients had undergone either a CT (*n* = 16) or MRI (*n* = 3) prior to EUS. 

**Figure 1:**
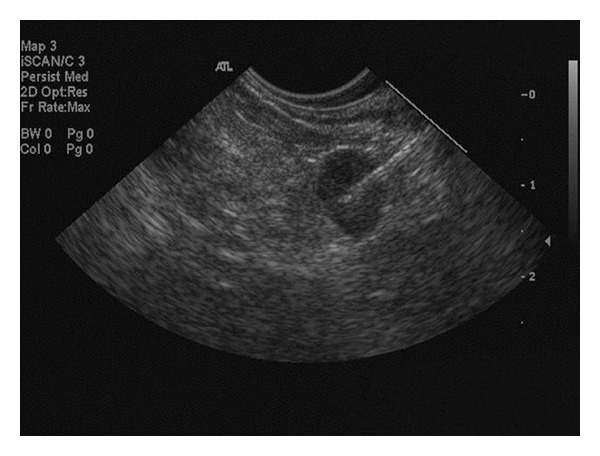
A round, hypoechoic, well-defined lymph node with a 7 mm short axis morphologically similar to any perirectal lymph node suggestive of malignancy but requiring FNA for clarification.

Seventeen patients (89%) had a prior urological cancer diagnosis at a median of 21.4 months (0–188 months) prior to EUS. Such cases included bladder (*n* = 10), prostate (*n* = 6), and testicular cancer (*n* = 1). Age, prior urological cancer diagnosis (stage where available), referral source, EUS FNA or TCB location, targeted biopsy or incidental finding, cytology ± immunostaining findings, and the final clinical diagnosis are provided in Table 1. The perirectal space (*n* = 6; 31.6%) was the most frequently sampled disease location irrespective of the primary urological cancer origin. The perirectal space was sampled to evaluate the presence of an extramurally located hypoechoic, irregular, and infiltrative process. Such lesions were not felt to represent lymph nodes or defined organs, or structures. The lymph nodes in the setting of prostate cancer were located proximal to the gland and were morphologically similar to any pelvic lymph node seen at EUS, traditionally primarily in the rectal cancer setting (Figure 1). Local recurrence like a rectal cancer local recurrence seemed to be subepithelial in the wall or adjacent to the rectal wall. Extramural drop metastases are also a possibility not recognized to our knowledge within this limited cohort of nineteen patients. 

Two patients had no prior urological cancer diagnosis, including a 64-year-old patient in whom initial CT revealed a 7.7 cm necrotic appearing right pelvic mass invading the rectum that was felt to arise from either the prostate or the bladder. EUS imaging alone could not decipher the primary tumor site but an FNA of the perirectal space revealed a poorly differentiated carcinoma consistent with an urothelial primary. Secondly, a 54-year-old patient with a history of hematuria, was found on initial CT and MRI to have a 2.5 cm low-density mass located adjacent to the left seminal vesicle. As part of the evaluation for possible prostate cancer, the referring urologist requested EUS FNA of the abnormal appearing seminal vesicle as part of the staging algorithm. EUS revealed a well-defined, partially cystic, nonvascular structure located adjacent to the left lobe of the prostate. The resulting cytology revealed no evidence for malignancy and that the sampled structure represented the seminal vesicle. There has been no evidence of malignancy during 7 years of followup. 

A median of 3 FNA passes (range 1–9) were performed revealing a sensitivity, specificity, PPV, and NPV of 94.4% (72–99), 100% (2–100), 100% (80–100), and 50% (1–98), respectively. Three patients underwent EUS TCB, 2 of which had been previously reported by our group [10]. The third patient was a 38-year-old male, who, 11 years previously, had a right orchiectomy for a testicular germ cell tumor, no chemotherapy and was under clinical surveillance, normal alpha fetoprotein level and HCG level was <2 IU/L. The accuracy and precision of this HCG assay is poor below 2.0 IU/L and therefore not useful in this particular case. He represented with symptomatic anemia and a 6.4 cm duodenal wall mass on CT. EUS revealed a circumferential ulcerated mass involving the 2nd and 3rd portion of the duodenum. Cytological review was positive for malignancy, but the histological review of the TCB specimens established a metastatic nonseminomatous germ cell tumor consistent with an embryonal carcinoma. Seven (37%) patients died during a 19.5 (0.5–87) months follow-up period. 

**Figure 2:**
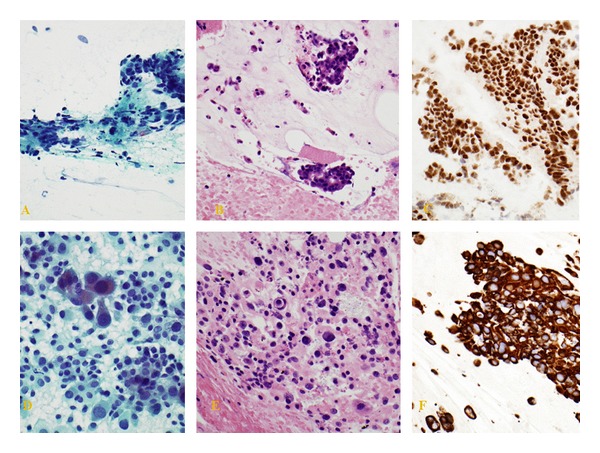
Distinct bladder wall and rectal wall FNA sites revealing high-grade urothelial carcinoma and adenocarcinoma, respectively, synchronously in the same patient. (A) Rectal wall: positive for malignancy. Adenocarcinoma consistent with colorectal primary. (Pap Stain); (B) rectal wall: positive for malignancy. Adenocarcinoma consistent with colorectal primary (H&E stain); (C) rectal wall: neoplastic cells are positive for CDX2 immunostaining; (D) bladder wall: positive for malignancy. High-grade urothelial carcinoma. (Pap Stain); (E) bladder wall: positive for malignancy. High-grade urothelial carcinoma. (H&E stain); (F) bladder wall: CK903 positive neoplastic cells with immunostaining.

One patient (5.2%) developed a procedure-related complication. Gross hematuria developed following FNA of a bladder mass in an 82-year-old male with a history of a transitional cell bladder cancer that had been managed by a transurethral resection of the tumor and 6 cycles of intravesical BCG. The patient was undergoing a lower GI EUS to evaluate a newly diagnosed rectal adenocarcinoma (T2). During EUS, an unsuspected bladder wall mass was identified which endosonographically, did not communicate with the rectal mass. Following consultation with the referring physician, an FNA of the bladder wall mass was performed revealing a high-grade urothelial carcinoma (positive immune-staining for CK7, CK20, and CK903). A rectal wall FNA was also performed, which was consistent with a separate primary rectal cancer based on positive CDX2, CK20, and CD903 staining and negative CK7 staining (Figures 2(A)–2(F)). Gross hematuria developed 12-hours after FNA and persisted for 48 hours without need for surgical intervention. The rectal cancer was managed by an ultralow anterior resection with end colostomy. The bladder recurrence was resected without evidence of invasion. The patient, subsequently, underwent a second induction course of BCG combined with interferon and had a negative cystoscopy three months later. 

## 4. Discussion

Cystoscopy alone is the most cost-effective strategy to detect superficial (noninvasive) bladder-cancer recurrence. The most common site of metastatic disease is locoregional lymph nodes that are reported in approximately one-third of surgical series [17, 18]. FDG-PET/CT may help to make treatment decisions prior to radical cystectomy as 17% of patients with a negative conventional preoperative evaluation have occult metastatic disease [19]. The limitations of CT and MRI nodal staging have encouraged the application of new technologies such as VEGF-C protein immunohistochemical staining to enhance nodal staging accuracy [20]. In this case series, we demonstrated the ability of EUS to identify otherwise unsuspected iliac vessel nodal disease in bladder cancer, a site beyond what can be identified with a traditional rigid transrectal ultrasound. 

The majority of prostate cancer recurrences following radical prostatectomy or radiation therapy are asymptomatic. Serum PSA is the noninvasive current gold standard to detect biochemical recurrence, but serum levels can fluctuate. Diagnostic PSA recurrence values are based on whether or not the patient had prior radiation therapy or not. Therefore, more invasive evaluations are required. But, the traditional methods for detecting local recurrence of prostate cancer (DRE or DRE plus TRUS or TRUS guided biopsy) provide poor diagnostic accuracy, and therefore have limited ability to guide salvage therapy. Digitally guided biopsy of a palpable abnormality and TRUS-guided biopsy have proven unreliable or necessitate repeated biopsy to establish local disease recurrence [21]. MRI becomes even less successful at detecting evidence of tumor recurrence following radiation therapy, cryosurgery, or high-intensity focused ultrasound. The site of disease recurrence after radical prostatectomy is a critical issue as it will influence the subsequent therapeutic strategy and patient management. Magnetic resonance lymphography has been recently reported to detect positive aberrant lymph nodes in the paraaortic, proximal common iliac, perirectal, and perivesical regions, which are outside the standard field for pelvic irradiation in 79% of patients with biochemical recurrence following radical prostatectomy [22]. In our limited experience, EUS was capable of identifying unsuspected perirectal lymph node involvement as well as tumor infiltration within the perirectal space as patterns of disease recurrence.

In conclusion, EUS-guided sampling of lymph nodes, the GI luminal wall, and perirectal space may increase the initial staging accuracy for urological cancers and may also enhance detection of local recurrence. These findings have the potential to significantly impact patient care and outcomes. EUS FNA of the aforementioned sites has been proven to be safe in multiple large series for nonurological pathology evaluations. However, there are few data concerning EUS-guided bladder FNA, for which one of our patients experienced delayed gross hematuria. In contrast to bladder biopsy during cystoscopy, during which immediate and targeted therapy can be applied, EUS does not facilitate this therapeutic option.

## Figures and Tables

**Table 1:** Clinical and EUS features of patients with primary disease, local recurrence or extra pelvic metastatic urological disease.

	Age & gender	Prior cancer diagnosis	Referral source	FNA location	Targeted biopsy or incidental finding	FNA passes	Cytologic interpretation	Immuno-staining^1^	Final diagnosis
1	84 M	Bladder	General surgery	Iliac LN	Targeted biopsy	5	Positive	CK7, CK20, OSCAR, p63	Recurrent bladder
2	82 M	Bladder (T1)	Colorectal surgery	Bladder mass	Incidental	3	Positive	CK7, CK20, CK903	Recurrent bladder
3	82 M	Bladder G3 muscle invasive	Urology	Perirectal space	Targeted biopsy	3	Positive	CK7, CK20	Recurrent bladder
4	64 M	No	Urology	Perirectal space	Targeted biopsy	1	Positive	NA	Bladder cancer
5	68 M	Prostate (T3b) & perianal Crohns disease	Urology	Perirectal space	Targeted biopsy	3	Negative	NA	Recurrent prostate following surgical evaluation
6	66 M	Prostate Gleason 3 + 3	Urology	Perirectal LN	Targeted biopsy	3	Positive	PSA, PACP	Recurrent prostate
7	77 M	Prostate Gleason 4 + 4	Urology	Perirectal LN	Targeted biopsy	5	Positive	PSA, PACP	Prostate cancer
8	95 M	Bladder G1	Gastroenterology	Perirectal LN	Targeted biopsy	3	Positive	PSA	Prostate cancer
9	60 M	Bladder	Gastroenterology	Rectal wall	Targeted biopsy	3	Suspicious	NA	Recurrent bladder³
10	53 M	Prostate Gleason 3 + 3	Urology	Perirectal space	Targeted biopsy	2	Positive	PSA, PACP	Recurrent prostate
11	73 M	Bladder G2 superficial	Urology	Rectal wall	Targeted biopsy	5	Positive	NA	Recurrent bladder³
12	54 M	Bladder G3 muscle invasive	Gastroenterology	Rectal wall	Targeted biopsy	3 & TCB (4)	Positive	CK7, CK20	Recurrent bladder³
13	54 M	No	Urology	Perirectal space	Targeted biopsy	4	Negative	NA	left seminal vesicle
14	55 M	Bladder G3 muscle invasive	Urology	Rectal wall	Targeted biopsy	2 & TCB (2)	Positive	CK7, CK20, CK903	Recurrent bladder³
15	70 F	Bladder G3 muscle invasive	Urology	Rectal wall	Targeted biopsy	9	Positive	Keratin AE1/AE3	Recurrent bladder
16	86 M	Prostate	Urology	Perirectal space	Targeted biopsy	2	Positive	NA	Recurrent prostate
17	40 M	Bladder G3 muscle invasive	Urology	Mediastinal LN	Targeted biopsy	4	Positive	NA	Metastatic bladder
18	37 M	Testicular embryonal and yolk sac tumor	Urology	Duodenal wall	Targeted biopsy	5 & TCB (3)	Positive	NA	Metastatic nonseminomatous germ cell tumor consistent with embryonal carcinoma
19	72 M	Prostate Gleason 3 + 3	Medical oncology	Subcarinal LN	Targeted biopsy	3	Positive	PSA, PACP^°^	Metastatic prostate

^1^CK7, 20, 903: Cytokeratin 7, 20, 903.

OSCAR: Monoclonal Antibody against Cytokeratin.

PSA: Prostate-specific antigen.

PACP: Human prostatic acid phosphatase.

Keratin AE1/AE3: detects CK1–8, 10, 14–16 and 19.

^°^Negative for CK7, TTF-1 and CDX2.

²LN: lymph node.

³Linitis plastica.

## Abbreviations

EUS: Endoscopic ultrasound
FNA: Fine needle aspiration
TCB: Tru-Cut biopsy.

## References

[1] Malmström PU (2005). Lymph node staging in prostatic carcinoma revisited. *Acta Oncologica*.

[2] Jensen TK, Holt P, Gerke O (2011). Preoperative lymph-node staging of invasive urothelial bladder cancer with 18F-fluorodeoxyglucose positron emission tomography/computed axial tomography and magnetic resonance imaging: correlation with histopathology. *Scandinavian Journal of Urology and Nephrology*.

[3] Häcker A, Jeschke S, Leeb K (2006). Detection of pelvic lymph node metastases in patients with clinically localized prostate cancer: comparison of [18F] fluorocholine positron emission tomography-computerized tomography and laparoscopic radioisotope guided sentinel lymph node dissection. *Journal of Urology*.

[4] Hövels AM, Heesakkers RAM, Adang EM (2008). The diagnostic accuracy of CT and MRI in the staging of pelvic lymph nodes in patients with prostate cancer: a meta-analysis. *Clinical Radiology*.

[5] Artifon ELA, Sakai P, Ishioka S (2007). EUS for locoregional staging of prostate cancer-a pilot study. *Gastrointestinal Endoscopy*.

[6] Artifon EL, Srougi M, Lucon AM, Sakai P, Bhutani MS (2009). Endoscopic ultrasound with fine-needle aspiration facilitates diagnosis of metastatic iliac lymph node invasion in prostate cancer. *Endoscopy*.

[7] Perez NE, Maryala S, Seren S, Feng J, Pansare V, Dhar R (2007). Metastatic prostate cancer presenting as mediastinal lymphadenopathy identified by EUS with FNA. *Gastrointestinal Endoscopy*.

[8] Bhutani MS (1999). EUS and EUS-guided fine-needle aspiration for the diagnosis of rectal linitis plastica secondary to prostate carcinoma. *Gastrointestinal Endoscopy*.

[9] Eloubeidi MA, Varadarajulu S, El-Galley R, Bueschen AJ, Eltoum I (2006). EUS-guided FNA for the diagnosis of recurrent bladder cancer through the ileal conduit: a novel approach. *Gastrointestinal Endoscopy*.

[10] Gleeson FC, Clain JE, Rajan E (2008). Secondary linitis plastica of the rectum: EUS features and tissue diagnosis (with video). *Gastrointestinal Endoscopy*.

[11] Varadarajulu S, Lee YT (2009). EUS 2008 Working Group document: evaluation of EUS-guided drainage of pelvic-fluid collections (with video). *Gastrointestinal Endoscopy*.

[12] Giovanni M, Bories E, Moutardier V (2003). Drainage of deep pelvic abscesses using therapeutic echo endoscopy. *Endoscopy*.

[13] Varadarajulu S, Drelichman ER (2007). EUS-guided drainage of pelvic abscess (with video). *Gastrointestinal Endoscopy*.

[14] Puri R, Eloubeidi MA, Sud R, Kumar M, Jain P (2010). Endoscopic ultrasound-guided drainage of pelvic abscess without fluoroscopy guidance. *Journal of Gastroenterology and Hepatology*.

[15] Yang J, Abdel-Wahab M, Ribeiro A (2009). EUS-guided fiducial placement before targeted radiation therapy for prostate cancer. *Gastrointestinal Endoscopy*.

[16] Yang J, Abdel-Wahab M, Ribeiro A (2011). EUS-guided fiducial placement after radical prostatectomy before targeted radiation therapy for prostate cancer recurrence. *Gastrointestinal Endoscopy*.

[17] Shinagare AB, Ramaiya NH, Jagannathan JP, Fennessy FM, Taplin ME, Van Den Abbeele AD (2011). Metastatic pattern of bladder cancer: correlation with the characteristics of the primary tumor. *American Journal of Roentgenology*.

[18] Kitamura H, Takei F, Nishida S, Muranaka T, Masumori N, Tsukamoto T (2012). Lymph node metastasis mapping in extended lymphadenectomy to the level of the inferior mesenteric artery for bladder cancer. *International Journal of Clinical Oncology*.

[19] Kibel AS, Dehdashti F, Katz MD (2009). Prospective study of [18F]fluorodeoxyglucose positron emission tomography/computed tomography for staging of muscle-invasive bladder carcinoma. *Journal of Clinical Oncology*.

[20] Li Z, Qi F, Miao J (2010). Vascular endothelial growth factor-C associated with computed tomography used in the diagnosis of lymph node metastasis of bladder carcinoma. *Archives of Medical Research*.

[21] Roscigno M, Scattoni V, Raber M (2002). The role of transrectal ultrasound of the prostatic fossa in the diagnosis of local recurrence after radical prostatectomy in case of PSA failure. *Archivio Italiano di Urologia e Andrologia*.

[22] Meijer HJM, van Lin EN, Debats OA (2011). High occurrence of aberrant lymph node spread on magnetic resonance lymphography in prostate cancer patients with a biochemical recurrence after radical prostatectomy. *International Journal of Radiation Oncology, Biology, Physics*.

